# Translational Animal Models Provide Insight Into Mesenchymal Stromal Cell (MSC) Secretome Therapy

**DOI:** 10.3389/fcell.2021.654885

**Published:** 2021-03-19

**Authors:** Rebecca M. Harman, Charlotte Marx, Gerlinde R. Van de Walle

**Affiliations:** Baker Institute for Animal Health, College of Veterinary Medicine, Cornell University, Ithaca, NY, United States

**Keywords:** mesenchymal stromal cells, stem cells, secretome, human, veterinary, translational models

## Abstract

The therapeutic potential of the mesenchymal stromal cell (MSC) secretome, consisting of all molecules secreted by MSCs, is intensively studied. MSCs can be readily isolated, expanded, and manipulated in culture, and few people argue with the ethics of their collection. Despite promising pre-clinical studies, most MSC secretome-based therapies have not been implemented in human medicine, in part because the complexity of bioactive factors secreted by MSCs is not completely understood. In addition, the MSC secretome is variable, influenced by individual donor, tissue source of origin, culture conditions, and passage. An increased understanding of the factors that make up the secretome and the ability to manipulate MSCs to consistently secrete factors of biologic importance will improve MSC therapy. To aid in this goal, we can draw from the wealth of information available on secreted factors from MSC isolated from veterinary species. These translational animal models will inspire efforts to move human MSC secretome therapy from bench to bedside.

## Introduction

Mesenchymal stromal cells (MSCs) are adult multipotent progenitor cells found in many organs and tissue types. Due to their relative ease of isolation and expansion in culture, combined with the lack of ethical constraints associated with the collection and manipulation of embryonic stem cells, MSCs hold great promise as a multi-faceted cell-based therapy (Pittenger et al., 2019). Originally considered as whole-cell therapy, whereby injected MSCs migrate to the site of tissue damage and differentiate into cells needed for repair or regeneration, it is now accepted that transplanted MSCs do not survive for long and that the effects of MSC-based therapies are due to a broad array of secreted bioactive factors, collectively referred to as the secretome (Maguire, 2013; Moll et al., 2020; Wu et al., 2020). The recognition that MSC secreted factors are responsible for the positive effects of MSCs on tissue repair is significant, as it spurs the design of MSC-based therapies that do not require administration of the cells themselves, thus avoiding negative immune reactions or unwanted tumor growth (Sun et al., 2019).

**FIGURE 1:**
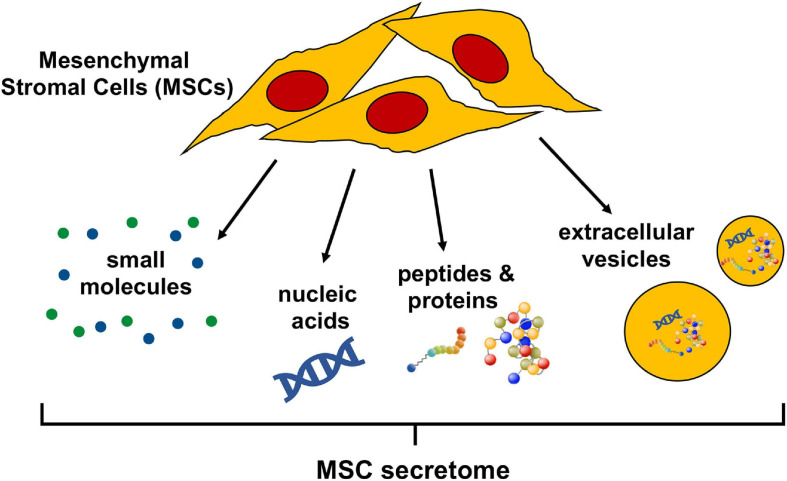
Bioactive components of the mesenchymal stromal cell (MSC) secretome. The MSC secretome is comprised of all factors secreted by MSCs. The bioactive components of the secretome include a wide range of small molecules, nucleic acids (importantly, regulatory RNAs), peptides, and proteins. These molecules can either be released freely or packaged in extracellular vesicles, which are lipid bilayer-delimited particles of various size and composition.

The secretome of cells in general, is a commixture of soluble factors as well as molecules associated with extracellular vesicles (EV); lipid bilayer delimited particles of various sizes and complexities containing proteins and nucleic acids released from cells into the extracellular space. Soluble factors, such as nucleic acids, proteins, and lipids, can all be detected in the cellular secretome, at various concentrations and activity levels determined by cell type and environment (Daneshmandi et al., 2020). The human MSC secretome is no exception and has been characterized as containing EV (Gowen et al., 2020), a multitude of regulatory non-coding RNAs (Harrell et al., 2019), as well as an abundance of proteins including growth factors, cytokines, peptides, and hormones (Abbasi-Malati et al., 2018). Lipid mediators are less well documented but have been described as active factors released by human MSC (Vasandan et al., 2016) (Figure 1). A solid understanding of the individual bioactive factors secreted by MSCs that affect injured target cells or tissues is indispensable to refine MSC secretome-based therapies and is not contradictory to evidence that cell free-based treatments greatly benefit from the administration of the complete secretome (Eleuteri and Fierabracci, 2019; Daneshmandi et al., 2020).

The MSC secretome has been actively explored for over 25 years (Haynesworth et al., 1996) but despite promising preliminary studies, no MSC secretome-based therapies are approved by the United States Food and Drug administration (FDA) for use in human medicine. This is in part because the secretome is not definitively characterized and typically varies significantly between MSC cultures dependent on individual donor, tissue source of origin, culture conditions, and passage (Rizk et al., 2016). To (i) help define the MSC secretome, (ii) understand which molecules secreted by MSCs are therapeutically valuable, and (iii) learn how to manipulate MSC to preferentially secrete these molecules, we can take advantage of information obtained from studies of MSCs isolated from veterinary species, many of which are relevant translational models for human conditions. The wealth of reports describing the activity of specific factors secreted by MSCs from veterinary species, as well the manipulation of these MSC to secrete factors of biological interest, will benefit human medicine by expanding the general knowledge of MSCs. And, by virtue of the fact that many veterinary species suffer from diseases that are physiologically analogous to human conditions that could be treated with MSC-secreted factors, they serve as relevant translational models.

This review starts with a brief overview of secreted factors from naïve human MSCs and laboratory rodent MSCs, which historically have been considered well-accepted animal models for human research. This is followed by a review of studies in which human or laboratory rodent MSCs were manipulated to improve inherent properties to optimize the therapeutic benefits of MSC secreted factors. Next, we provide an extensive overview of studies on the secretome of naïve, as well as manipulated, MSCs isolated from veterinary species, which function as physiologically relevant translational models for human MSC secretome-based therapies but are less well accepted.

## The Secretome of Mesenchymal Stromal Cells (MSCs) From Humans and Laboratory Rodents

### Naïve Human MSCs

The secretome of human MSCs regulates a wide variety of physiological processes. The pioneering studies of the effects of these secreted factors on specific target cells or in experimental rodent models have been extensively reviewed elsewhere (Vizoso et al., 2017; Abbasi-Malati et al., 2018; Eljarrah et al., 2019; Harrell et al., 2019). To provide an overview of the research conducted on human MSC secreted factors without being redundant, Table 1 summarizes human MSC secretome studies that have been published in more recent years (2018–2020). The majority of these studies evaluates either the effects of the complete secretome or exosomes (EXOs), a class of EV. An advantage to delivering MSC-secreted factors as EXOs is that EXOs can cross physiological barriers *in vivo*, which makes them attractive as treatments for diseases in tissues with restricted drug access such as the retina or the central nervous system (CNS) (Alvarez-Erviti et al., 2011; Zhang et al., 2015). Many of the studies do not identify the specific MSC secreted factors that mediate the observed effects on target cells or tissues, which illustrates the difficulty of determining precisely which factors in the complex secretome are responsible for biological responses.

**TABLE 1:** Human MSC secretome components, targets, effects, and potential therapeutic uses.

**MSC source**	**Secretome components**	**Targets: effects**	**Therapeutic use**	**References**
Bone marrow	Complete secretome including VEGFC, TGF-β1, TGF-β2, GDF6	Secretome not tested with targets in a model system	Hematological malignancies	Baberg et al., 2019
Adipose	EV derived alpha-1-antitrypsin	*S. aureus, K. pneumoniae, P. aeruginosa*: microbicidal effect on gram negative bacteria	Pulmonary disease	Bari et al., 2019
Umbilical cord, dental pulp	Complete secretome	HUVEC: decreased apoptosis and senescence, increased migration, tube formation, *in vitro* vascularization	General MSC based therapies	Caseiro et al., 2019
Umbilical cord	TSG-6 in complete CM, EXO	Newborn mouse model of BPD: improvement of lung, cardiac, and brain pathology	Bronchopulmonary dysplasia	Chaubey et al., 2018
Adipose	Complete secretome	Arsenic injured human neurons: prevent arsenic induced damage	Prevention of arsenic induced toxicity	Curtis et al., 2018
Hoffa’s fat pad, synovial membrane, umbilical cord, cartilage	Complete secretome including MMPs, IL-17, complement factors, TGF-β1 and PGE2	-Human PBMC: inhibition of proliferation, migration and cytokine secretion -Human chondrocytes: increased aggrecan gene expression	Articular cartilage repair	Islam et al., 2019
Adipose, bone marrow, Wharton’s jelly	Complete secretome	-Human monocyte: increased migration -Human macrophage: increased differentiation -Human endothelial cells: induced pro-angiogenic phenotype -Murine vasculature: increased vascularization in Matrigel plug assay	Ischemic diseases	Kehl et al., 2019
Bone marrow	Complete secretome including IL-5, IL-6, IL-8, IL-9, IP-10 MCP-1, FGF-2 and VEGF	Human keratinocytes in hypoxic, low serum culture: increased migration and proliferation, cell spreading and F-actin expression	Chronic wounds	Kosol et al., 2020
Umbilical cord	Complete secretome	-Rat bone marrow MSC from aged animals: increased cell growth, differentiation, potential, decreased senescence -Aged rats: improved bone formation capacity	Age-related osteoporosis	Liang et al., 2019
Bone marrow	miR-21-5p from EXO	Human engineered cardiac tissue: increased contractility, calcium handling	Cardiac therapies	Mayourian et al., 2018
Bone marrow	Complete secretome including EXO-related proteins related to the ubiquitin-proteosome and histone systems	Human neural progenitors: induced neural differentiation Rat model of Parkinson’s disease: rescued dopamine neurons, increased behavioral performance in staircase test	Parkinson’s disease	Mendes-Pinheiro et al., 2019
Adipose	Complete secretome, soluble factors and EV cargo including proteins involved in RNA metabolism and miRNAs targeting processes involved in regeneration, regulation of inflammation	-Human and rat cell lines: increased proliferation and differentiation, protection against senescence -Mouse model of skeletal muscle injury: enhanced rate of regeneration after acute damage	Muscle regeneration	Mitchell et al., 2019
Adipose	Complete secretome including TIMPs and cartilage protecting factors	TNFα-stimulated primary articular chondrocytes: blunted hypertrophy, reduced levels of osteocalcin and collagen X and MMP13 activity	Osteoarthritis	Niada et al., 2019
Adipose	Innate and IFNγ preconditioned/complete secretome including > 60 secreted cytokines/chemokines and >240 EV-miRNAs	-Macrophages: increased anti-inflammatory phenotype marker CD163 -Chondrocytes: reduced inflammation marker VCAM1	Joint disease	Ragni et al., 2020
Cornea	Complete secretome	*Ex vivo* porcine cornea injury model: enhanced survival of corneal endothelial cells	Corneal endothelial cell injury	Rouhbakhshzaeri et al., 2019
Adipose	Concentrated secretome including GDNF and FGF2	Rat model of bilateral abdominal cryptorchidism: restored seminiferous tubules, increased *GATA4* expression	Non-obstructive spermatogenesis disorders	Sagaradze et al., 2019
Cornea	EXO	-Cultured corneal epithelial cells: increased migration -Murine epithelial debridement wounds: increased wound healing	Ocular surface injuries	Samaeekia et al., 2018
Bone marrow, adipose	Complete secretome	Hypoxic primary rat alveolar epithelial cells: increased viability, reduced secretion of inflammatory mediators, enhanced IL-10 production, increased active MMPs	Pulmonary syndromes	Shologu et al., 2018
Wharton’s jelly, bone marrow	EXO	Mouse model of bronchopulmonary dysplasia: ameliorated alveolar simplification, fibrosis and pulmonary vascular remodeling due to hyperoxia	Pulmonary disease	Willis et al., 2018

*BPD, bronchopulmonary dysplasia; EV, extracellular vesicle; EXO, exosomes; FGF2, fibroblast growth factor 2; GATA4: GDF6, growth differentiation factor 6; HUVEC, human umbilical vein endothelial cells; IFNγ, interferon γ; IL, interleukin; IP-10, interferon γ -induced protein 10; MCP-1, monocyte chemoattractant protein-1; MMPs, matrix metalloproteases; PBMC, peripheral blood mononuclear cells; PGs, prostaglandins; PGE2, prostaglandin E2; TIMPs, tissue inhibitors of MMPs; TGF, transforming growth factor; TSG-6, tumor necrosis factor α-stimulated gene-6; VEGF, vascular endothelial growth factor; VECAM1, vascular cell adhesion protein 1; VEGFC, vascular endothelial growth factor C.*

### Naïve Rodent MSCs

As relatively inexpensive, well-accepted models for human disease, rats and mice have been used for evaluating MSC-based therapies for human diseases. Of the many published rodent MSC studies, Table 2 gives an overview of recent work (2013–2018) focused specifically on MSC secretome components. As with the human studies, the results tend to promote the utility of the entire secretome and/or or EXOs, as opposed to confirming the specific molecules responsible for the observed biological effects.

**TABLE 2:** Rodent MSC secretome components, targets, effects, and potential therapeutic uses.

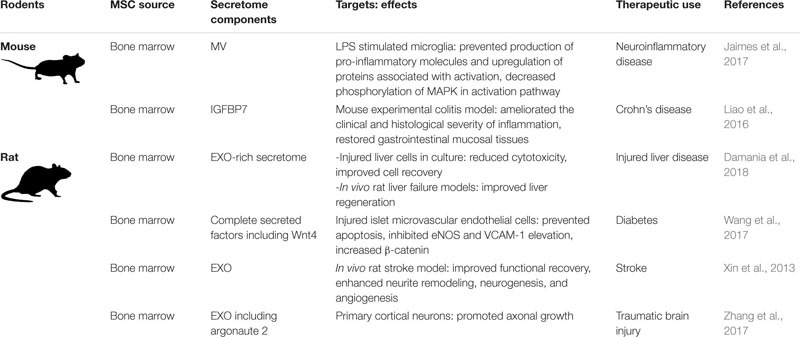

*eNOS, endothelial nitric oxide synthase; EXO, exosome; IGFBP7, insulin-like growth factor binding protein 7; LPS, lipopolysaccharide; MAPK, mitogen-activated protein kinase; MV, microvesicles; VCAM-1, vascular cell adhesion protein 1.*

### Manipulated MSCs

Although it is clear that MSCs have great therapeutic potential and are being explored in multiple medical fields, results are often inconsistent and (pre-)clinical studies show only minor effects or do not lead to the desired outcomes at all (Lukomska et al., 2019; Wilson et al., 2019). A potential reason for the observed inconsistencies is the heterogeneity of cultured MSCs, which is influenced by the individual donor and tissue of origin, isolation technique, culture environment, and cell passage number (Wilson et al., 2019; Harman et al., 2020). Purposefully manipulating MSCs to improve therapeutic benefits, which by default could result in a more standardized and/or customized secretome, is not a novel idea, but one that has not yet been maximally explored (Liesveld et al., 2020; Ocansey et al., 2020).

**FIGURE 2:**
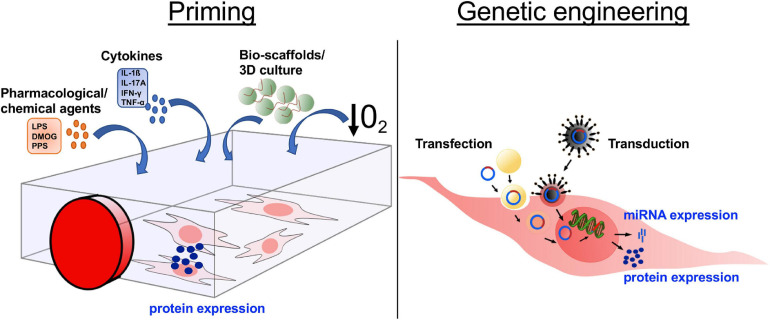
Altering the mesenchymal stromal cell (MSC) secretome through *in vitro* manipulation. In order to increase the secretion of desired molecules, such as proteins or miRNA, MSCs are manipulated in culture through either priming or genetic engineering. The four main approaches of priming are (i) addition of pharmacological/chemical agents, (ii) treatment with cytokines, (iii) culture in 3D cultures/bio-scaffolds, and (iv) culture under induced hypoxic conditions. Genetic engineering is used to express/overexpress specific proteins or miRNA by a targeted RNA or DNA transfer into the MSCs via transduction, transfer by virus or viral vector, or transfection, transfer through various biological/chemical/physical approaches. DMOG, dimethyloxalylglycine; LPS, lipopolysaccharides; PPS, pentosan polysulfate; IL, interleukin; IFN, interferon; TNF, tumor necrosis factor; 3D, 3-dimensional; O_2_, oxygen.

The methods by which MSCs are manipulated to control secretome components broadly fall into two categories*:* (i) priming and (ii) genetic modification (Figure 2). Priming MSCs to improve their immunomodulatory properties, migratory potential, and/or hypo-immunogenicity, has become a field of intense research. The most popular strategies for priming include treatment with pharmacological or chemical agents, stimulation with cytokines, alterations of culture conditions via use of bio-scaffolds and/or 3D cultures, and the use of hypoxic culture conditions (Figure 2). The various strategies to influence human MSC behavior via priming have been reviewed in detail recently (Noronha et al., 2019). Genetic modification of MSCs to silence or overexpress genes of interest via transfection and/or transduction is gaining increased attention (Figure 2). Transfection approaches include microinjection, electroporation, and nanocarriers (including polymers, lipids, polysaccharides, peptides, and inorganic materials) (Hamann et al., 2019). Transduction, using viral vectors such as lentivirus and adenovirus, has the advantage of being more efficient than transfection, however, safety concerns related to potential immunogenicity and mutagenicity of the viral vectors are disadvantages of this technique for clinical use. MSCs secrete miRNAs, which are short oligonucleotides with critical post-transcriptional regulatory functions that are either released within EV or protein-associated, where miRNAs are vesicle-free but associated with high-density lipoproteins or Argonaute-2/nucleophosmin-1 (Chen et al., 2012). Overexpressing or inhibiting miRNAs in MSCs is considered a valuable approach to improve clinical outcome (He and Hannon, 2004; Yeo et al., 2013) (Figure 2).

A potential, albeit minimally explored at present, method of manipulating the MSC secretome is via the exogenous bioengineering of isolated EVs. Approaches to bioengineer EVs secreted from various cell types and their therapeutic applications are the topic of a recent review (Wiklander et al., 2019). While these methods have not been widely used for MSC-derived EVs, they could become an interesting approach to control the composition of bioactive factors in the MSC secretome.

#### Manipulated Human MSCs

The manipulation of human MSCs to enhance secretion of desired factors, to increase homing abilities, and/or to decrease their immunogenicity, has been reviewed in depth by other authors (Najar et al., 2019; Costa et al., 2020; Ullah et al., 2019). Here, we will give a short overview of recent studies in which human MSCs have been manipulated either by priming or genetic engineering (Table 3). Of note is that studies of human MSCs are usually conducted *in vitro*. In order to investigate the effects of manipulated MSC *in vivo*, researchers typically fall back on laboratory rodent models, as outlined below, raising the question of direct translation from mice to men. As an exception, pilot studies and clinical trials have been conducted in humans using MSC for gene-directed enzyme/prodrug therapy (GDEPT) (von Einem et al., 2017, 2019). With the GDEPT approach, MSCs are solely used as vehicles to carry cargo, which consists of enzymes capable to activate the prodrug form of chemotherapeutics, into tumors. The fact that the administered inactive prodrug gets converted by the enzymes at the tumor site offers the advantage that the complete anti-tumor effect of the chemotherapeutic only unfolds locally without causing severe side effects systemically (Matuskova et al., 2015).

**TABLE 3:** Manipulation of human MSCs to optimize the therapeutic effects of the MSC secretome.

**Modification (MSC source)**	**Manipulation**	**Outcome**	**Therapeutic use**	**References**
**Priming**				
Protein profile in EXO (BMMSC)	Retinal cell CM (TNF-α)	MSC CM and EXO had neuro-protective effects on retinal ganglion cells, increased PEDF and VEGF-A in primed EXO	Optic nerve injury	Mead et al., 2020
Immune-modulatory properties (Gingival MSC)	IL-1β	Overexpression of TGF-β and MMP pathway agonists (MMP-1, MMP-9), Primed MSC CM promoted cell migration, epidermal-dermal junction formation, inflammation reduction *in vitro* and improved epidermal engraftment *in vivo* (mice)	Wound healing	Magne et al., 2020
Protein profile in EXO (BMMSC)	Hypoxia	Exosomes from primed MSC are enriched with specific subclassifications of proteins, including secretory and ECM associated proteins, EXO enhances secretion of growth factors of neuroblast-like cells	CNS related diseases	Yuan et al., 2019
Immune-modulatory properties (UCMSC)	3D culture	CM had increased anti-inflammatory profile (IL-10, LIF) and trophic factors (PDGF-BB, FGF-2, I-309, GM-CSF, increased therapeutic effect *in vivo* (rats)	Rheumatoid arthritis	Miranda et al., 2019
Senescence and immune-modulatory properties (BMMSC)	Substance P	Increased secretion of PDGF-BB in primed MSC. Primed MSC CM increased viability of retinal pigmented epithelium	Age related macular degeneration	Jung et al., 2019
Metabolic pathways (BMMSC)	INF-γ/TNF-α	Primed MSC show increased glycolysis and fatty acid oxidation, glycolysis is linked to MSC-mediated T cell suppression through the JAK/STAT1/IDO axis by posttranslational modification (glycosylation) of STAT1	General MSC therapy: immune-modulatory properties	Jitschin et al., 2019
Induction of quiescent state (BMMSC)	Hypoxia, SF-media	Increased survival, adaptive response mechanism after transplantation, primed MSC maintained their stemness by reaching a quiescent state.	General MSC therapy	Ferro et al., 2019
Senescence and immune-modulatory properties (BMMSC)	3D culture in FBS-containing medium and xeno-free medium	MSC in 3D culture contained their immune-suppressive profile over multiple passages. Upregulation of COX-2, TNF alpha induced protein 6, SCT-1. Secretion of PGE_2,_ TSG-6, STC-1	General MSC therapy: immune-modulatory properties	Bartosh and Ylostalo, 2019
**Genetic modification: miRNA overexpression**				
miRNA-26a-5p (BMMSC)	Lentivirus	Alleviation of damages on synovial fibroblasts by targeting PTGS2 *in vitro* and retardation of damage in OA *in vivo* (rats)	Osteoarthritis	Jin et al., 2020
miRNA-181a (UCMSC)	Lentivirus	Reduced inflammatory response and promoted Treg polarization *in vitro* and an *in vivo* (mice) ischemic damage model	Myocardial infarction	Wei et al., 2019
miRNA-122 (ADMSC)	Lipofection	Reduced collagen, inhibition of pro-inflammatory cytokines, reduction of liver enzymes, elevated expression of antifibrotic proteins *in vitro* (human HSC) and *in vivo* (mice)	Liver fibrosis	Kim K.H. et al., 2019
miRNA-126 (UCMSC)	Lipofection	Alleviated effects of hypoglycemia induced inflammation *in vivo* (rats), suppressing of HMGB-1 signaling pathway and inflammation *in vitro* (human retina cells)	Retinal inflammation in diabetes	Zhang et al., 2019
**Genetic modification: protein overexpression via transfection**				
VEGF (BMMSC)	Microporation	Improved angiogenic potential *in vitro*	Peripheral artery disease	Serra et al., 2019
CXCR4 (BMMSC, ADMSC)	Microporation in combination with minicircle transfection	Increased homing in a skin wound mouse model	General MSC therapy: homing	Mun et al., 2016
**Genetic modification: Transduction of viral immune evasion proteins**				
Herpesviral immunoevasion protein US11 (BMMSC)	Lentivirus	Downregulation of MHCI proteins, increased persistence of MSC in immune-competent mice with depleted NK	General MSC therapy/increase of immune evasiveness	de la Garza-Rodea et al., 2011
Cytomegaloviral immunoevasion proteins US6/US11 (not specified)	Retrovirus	Downregulation of HLA-I, protection against NK in vitro, increased liver engraftment in pre-immune fetal sheep	General MSC therapy/increase of immune evasiveness	Soland et al., 2012
**Genetic modification: Gene-directed enzyme/prodrug therapy**				
HSVtk (BMMSC)	Retrovirus	Clinical trial, administration of transduced MSC in combination with prodrug ganciclovir: 4 of 6 patients reached stable disease, safe and feasible	Gastrointestinal adenocarcinoma	von Einem et al., 2017
HSVtk (BMMSC)	Retrovirus	Clinical trial, administration of transduced MSC in combination with prodrug ganciclovir: 50% of patients reached stable disease, safe and feasible	Gastrointestinal adenocarcinoma	von Einem et al., 2019
CD::UPRT or HSVtk (ADMSC)		Systemic administration of human CD::UPRT-MSC or HSVtk-MSC in combination with 5-FC and ganciclovir inhibited growth of lung metastases in mice	Gastrointestinal adenocarcinoma	Matuskova et al., 2015

*AD, adipose tissue derived; BM, bone marrow derived; BNDF, brain-derived neurotrophic factor; CD, cluster of differentiation; CM, conditioned medium; CNS, central nervous system; COX, cyclooxygenase; EXO, exosomes; FBS, fetal bovine serum; FGF, fibroblast growth factor; GM-CSF, granulocyte-macrophage colony stimulating factor; HGF, hepatocyte growth factor; HLA, human leukocyte antigen; HSC, hepatic stellate cells; IDO, indoleamine-pyrrole 2,3-dioxygenase; IL, interleukin; INF, interferon; JAK, janus kinase; LIF, leukemia inhibitory factor; MHC, major histocompatibility complex; MMP, matrix metalloproteinase; MSC, mesenchymal stromal cells; NK, natural killer cells; OA, osteoarthritis; PEDF, pigment epithelial derived factor; PGE_2_, prostaglandin E_2_; PSGL, *P*-selectin Glycoprotein Ligand; PTGS2, prostaglandin-endoperoxide synthase 2; STC-1, stanniocalcin 1; SF, serum free; STAT, signal transducer and activator of transcription; TGF, transforming growth factor; TNF, tumor necrosis factor; TSG, tumor necrosis factor-stimulated gene; UC, umbilical cord derived; VEGF, vascular endothelial growth factor; HSVtk, herpes simplex thymidine kinase; CD::UPRT, fusion yeast *cytosine deaminase::uracil phosphoribosyltransferase*, 5-FC: 5-fluorocytosine.*

#### Manipulated Rodent MSCs

Laboratory rodent models are the gold standard for investigating the effects of manipulated MSCs *in vivo*. Advantages of these models are the relatively low cost, the availability of genetically modified animals designed to model human diseases, and the availability of commercially customized reagents for the use of research in rodents. Since it is common to use rodents for *in vivo* research, experimental work *in vitro* with optimized human MSCs is often followed up by related *in vivo* studies using rodent MSCs in experimentally induced disease models. Table 4 gives an overview of recent studies testing the efficacy of manipulated rodent MSC.

**TABLE 4:** Manipulation of laboratory rodent MSCs to optimize the therapeutic effects of the MSC secretome.

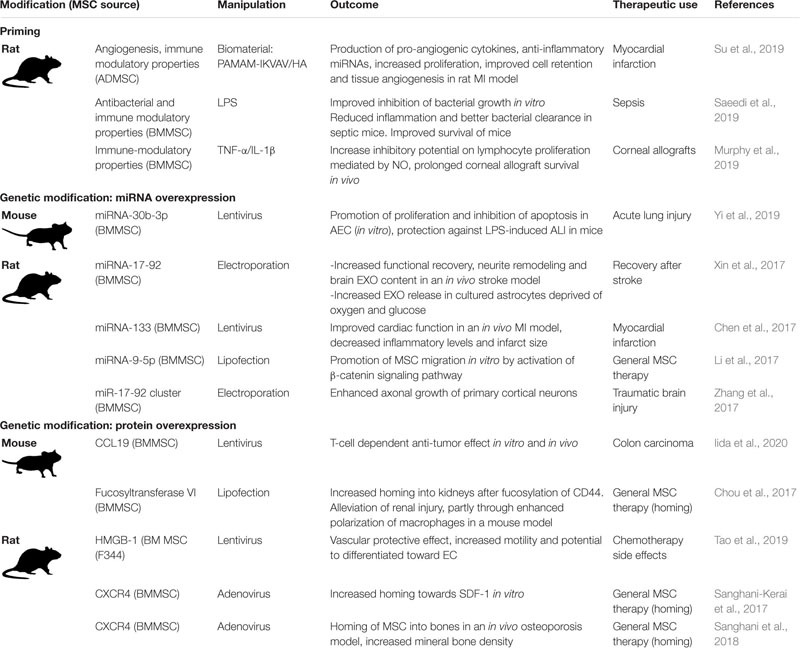

*AD, adipose tissue derived; AEC, alveolar epithelial cells; ALI, acute lung injury; BM, bone marrow derived; CD, cluster of differentiation; CM, conditioned medium; EC, endothelial cell; EXO, exosomes; HMGB1, high-mobility group box 1; IL, interleukin; INF, interferon; LPS, lipopolysaccharide; MI, myocardial infarct; MSC, mesenchymal stromal cells; NO, nitric oxide; PAMAM-IKVAV/HA, polyamidoamine-IKVAV/hyaluronic acid; PGE_2_, prostaglandin E_2_; SDF-1, stromal cell-derived factor 1; TNF, tumor necrosis factor; UC, umbilical cord derived.*

## The Secretome of Mesenchymal Stromal Cells (MSCs) From Veterinary Species

### Translational Potential of MSC Research in Veterinary Species

Mesenchymal stromal cells derived from veterinary species have been isolated, characterized and extensively studied *in vitro*, with the goal of determining the potential of these cells as therapies for a variety of diseases, many of which also affect humans (De Schauwer et al., 2013; Calloni et al., 2014; Devireddy et al., 2017; Sultana et al., 2018; Dias et al., 2019; Hill et al., 2019). In addition, case studies and clinical trials of MSCs isolated from companion animals have provided *in vivo* data further supporting the efficacy of MSC-based therapies (Caniglia et al., 2012; Carvalho et al., 2013; Renzi et al., 2013; Arzi et al., 2016; Hoffman and Dow, 2016; Geburek et al., 2017; Quimby and Borjesson, 2018). This section provides an overview of data from studies specifically designed to look at the effects of secreted factors of MSC isolated from veterinary species for diseases that are relevant to human medicine.

Veterinary species as physiologically relevant translational models for human diseases have several advantages over rodent models. When working with veterinary species MSC, results from *in vitro* experiments can be easily tested in the same species *in vivo*. In addition, most veterinary species (i) are made up of individuals with genetic variation that reflects the diversity found in human populations, (ii) have larger body sizes and longer lifespans compared to rodents, and (iii) are often exposed to the same environmental insults as humans, causing them to be susceptible to similar naturally occurring diseases, such as musculoskeletal disorders, immune-modulatory diseases, respiratory diseases, and certain cancers. As such, they serve as valuable “real world” models (Hoffman and Dow, 2016).

**FIGURE 3:**
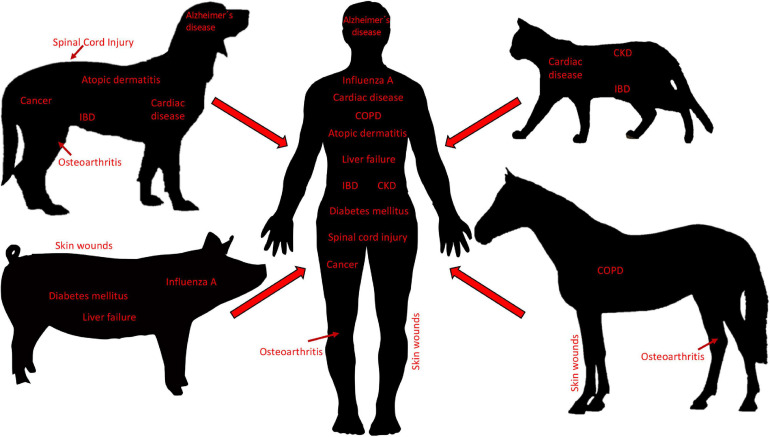
Diseases in veterinary species as translational models for human disease. Small companion animals (i.e., dogs and cats), as well as large animals (i.e., pigs and horses), develop pathologies that are similar to diseases in humans and thus, are used as translational animal models for neurological, cardiac, pulmonary, musculoskeletal, nephrological, gastroenterological, dermatological, infectious, and cancerous, diseases. Body sizes are not to scale. IBD, inflammatory bowel disease; CKD, chronic kidney disease; COPD, chronic obstructive pulmonary disease.

Small companion animals, most often dogs and cats, are domesticated animals whose physical, emotional, behavioral and social needs are met by close daily relationships with humans. Human bonds to companion animals create a demand for new and optimal pet therapies, including state-of-the-art cell-based therapies. Consequently, the growing interest in MSC-based therapies has resulted in MSCs from dogs and cats to be isolated, characterized, and studied in both the laboratory setting and clinical trials (Hoffman and Dow, 2016). Generally, the dog is considered an excellent model for human disease. In addition to sharing similar environments with humans, dogs naturally develop diseases that resemble pathologic conditions of humans (Hoffman and Dow, 2016). In this regard, MSC-based therapies have been widely investigated in diseases in dogs, including osteoarthritis (OA), spinal cord injury, bone regeneration, pulmonary and cardiac disorders, cancer, intervertebral disk degeneration, atopic dermatitis, inflammatory bowel disease (IBD), non-ischemic cardiac disease, Alzheimer’s disease, amyotrophic lateral sclerosis, and epilepsy (Hoffman and Dow, 2016). Cats have proven to be good models for immune-mediated diseases such as IBD as well as non-ischemic cardiac disease, and chronic kidney disease (Hoffman and Dow, 2016) (Figure 3).

Mesenchymal stromal cells have been isolated and characterized from large animals including cows, pigs, sheep, goats, and horses (Calloni et al., 2014). Of these, the majority of MSC studies have been carried out with MSCs isolated from horses, driven by the high demand of horse owners for innovative regenerative therapies, primarily geared toward the treatment of musculoskeletal injuries. Horses are well-characterized as models for specific human diseases, most notably orthopedic injuries such as OA (McCoy, 2015), but also skin wounds (Harman et al., 2019), and respiratory diseases (Klier et al., 2019), all of which have the potential to be managed by treatment with MSC secreted factors (De Schauwer et al., 2013) (Figure 3). Pigs have long been considered valuable preclinical models for a variety of human therapies, as exemplified by the use of pig organs that are quite similar to those of humans in terms of size, morphology, and function (Roth and Tuggle, 2015). Assessing stem cell-based therapeutics in pig models for skin wounds, acute liver failure, neurodegenerative disorders, general wound healing and tissue repair, diabetes mellitus, and influenza A infections, have all been proposed, and are mainly performed to fine-tune preclinical testing (Sullivan et al., 2001; Rajao and Vincent, 2015; Seaton et al., 2015; Bharti et al., 2016) (Figure 3). Moreover, there is evidence that pig MSCs can function cross-species *in vivo* (Li et al., 2014). Lastly, Sheep and goats are primarily used to model human OA (McCoy, 2015), and sheep also serve as models for human respiratory diseases (Meeusen et al., 2009). The sheep model has also been used to examine the therapeutic effects of EVs derived from human BM-MSCs in a preclinical model of hypoxic-ischemic brain injury in preterm neonates (Ophelders et al., 2016). In this study, ovine fetuses were subjected to global hypoxia-ischemia followed by *in utero* intravenous treatment with EVs. As compared to controls, brain function in fetuses treated with EVs exhibited improved brain function as determined by total number and duration of seizures and preserved baroreceptor reflex sensitivity. Although cerebral inflammation remained unaffected by this treatment, the authors proposed that MSC EVs might be a novel approach to reduce neurological consequences of hypoxic-ischemic injury of the fetal brain in humans (Ophelders et al., 2016).

It is important to point out that many MSC secreted factors are similar across species, making studies on the MSC secretome from animals relevant to human medicine. For example, immunomodulatory molecules, growth factors, anti-tumoral, and anti-microbial molecules, have all been documented to be secreted by MSCs isolated from humans, laboratory rodents, and veterinary species (Harman et al., 2017b; Vizoso et al., 2017; Cassano et al., 2018; Mancuso et al., 2019; Villatoro et al., 2019). Although a quantitative analysis of MSC secreted factors across species has not been carried out to our knowledge, such studies could further strengthen the translational aspect of MSC secretome studies in animals.

### Naïve Small Companion Animal MSCs

The primary source of MSC isolated from dogs and studied for therapeutic use is adipose tissue (AT). The secretome of AT-derived MSCs from dogs has the potential to influence neurologic diseases, immune-related diseases and cancer. *In vitro* experiments designed to examine the paracrine action of dog AT-derived MSCs on neuronal and endothelial cells showed that treating a neuronal cell line with conditioned medium (CM) from MSC cultures significantly increased cell proliferation, neurite outgrowth and expression of the neuronal marker βIII-tubulin (Al Delfi et al., 2016). Exposure of an endothelial cell line to this dog MSC CM increased cell proliferation and migration, as well as inducing tubule formation in a soluble basement membrane matrix, suggesting that the MSC secretome contains pro-angiogenic factors (Al Delfi et al., 2016). The authors concluded that these data support the hypothesis that transplanted MSC can promote increased neural function in dogs with CNS damage, due to paracrine activity on nerves and blood vessels (Al Delfi et al., 2016). Of note is that the neuronal and endothelial cell lines used in this study for the experiments were of human origin. The fact that dog-derived secretome components stimulated human cells suggests common inter-species mechanisms that endorse the use of veterinary models for human medicine. Another *in vitro* study aimed to compare immunomodulatory properties of dog AT- and bone marrow (BM)-derived MSCs. In these experiments, proliferation of stimulated peripheral blood mononuclear cells (PBMCs) from dogs co-cultured with AT- or BM-derived MSCs was inhibited when compared to non-co-cultured controls (Russell et al., 2016). The authors concluded that the *in vitro* immunomodulatory effects were mediated by MSC secreted factors and proposed future *in vivo* experiments to determine the efficacy of MSCs to modulate the immune system during inflammation-based conditions in dogs (Russell et al., 2016). Experiments testing the effects of dog AT-derived MSC CM on tumor cell growth showed that the MSC CM enhanced proliferation and invasion of a dog hepatocellular carcinoma cell (HCC) line *in vitro* and altered mRNA expression levels of genes related to tumor progression in HCC cells (Teshima et al., 2018). The results of this study seemingly contradicted multiple earlier studies on the effects of human MSC CM on human HCC cell lines, where inhibition of proliferation and invasion were observed (Li et al., 2010; Zhao et al., 2012). This indicates that more research is needed in both species to determine if and how the MSC secretome influences cancer cell growth and tumor progression (Teshima et al., 2018).

The secretome of AT-derived MSC from cats has been studied as well, particularly in the context of immune modulation. IBD is an autoimmune disease common in both cats and humans. *In vitro* experiments showed that prostaglandin E_2_ (PGE_2_) secreted by cat AT-MSC induced elevation of Forkhead box P3 (*FOXP3*) mRNA and altered the expression of inflammatory cytokines in concanavalin A (Con A)-stimulated PBMCs (An et al., 2018). Complementary studies in a Dextran sulfate sodium -treated mouse model of colitis demonstrated that intraperitoneal infusion of cat AT-MSCs reduced the clinical and histopathologic severity of colitis, and FOXP3+ T cells were significantly increased in the inflamed colon of MSC-treated mice as compared to controls (An et al., 2018). The authors concluded that PGE_2_ secreted by cat AT-MSC likely reduced inflammation by increasing FOXP3+ regulatory T cells in the mouse model and proposed that MSC-derived PGE_2_ may improve IBD and other immune-mediated inflammatory diseases in cats (An et al., 2018). Cat AT-MSC secreted factors have been shown to decrease proliferation of Con A-stimulated PBMCs, suggesting an additional anti-inflammatory mechanism (Parys et al., 2017). Inhibition of cat AT-MSC secreted PGE_2_ by indomethacin or NS-398 was shown to reduce the anti-proliferative effects of AT-MSC CM on cat PBMCs, confirming that PGE_2_ is involved in the immunomodulatory effects exerted by the MSC secretome (Chae et al., 2017; Taechangam et al., 2019). One of these studies also showed that cat AT-MSC secreted factors alter cytokine expression in cat PBMCs as well as a murine macrophage cell line, providing more evidence that inter-species studies of the MSC secretome can provide data that is relevant to human medicine (Chae et al., 2017). A more detailed comparison of the cat and human AT-MSC secretome using ELISAs and enzyme activity assays, revealed that AT-MSC from both species secrete PGE_2_, indoleamine 2,3 dioxygenase, transforming growth factor beta (TGFβ) and interleukin (IL)- 6, and that secretion of these proteins was increased when MSCs were co-cultured with stimulated PBMCs (Clark et al., 2017). Clinical trials using cats with naturally occurring inflammatory and immune-mediated diseases could, therefore, be used as surrogate models for human clinical trials (Clark et al., 2017). Supernatants from cat BM- and AT-derived MSC cultures have also been shown to modulate immune cells by inhibiting the reactive oxygen species (ROS) production by cat neutrophils *in vitro*. Although the authors of this study did not attempt to identify specific factors in the supernatants that were responsible for exerting this effect, it was determined to be dose dependent, with ROS production decreasing when neutrophils were cultured in medium made up of increasing percentages of MSC supernatants (Mumaw et al., 2015). Moreover, cat MSCs from both sources displayed similar effects on neutrophil ROS production, and the authors further concluded that supernatants from cat BM- and AT-derived MSC cultures could be clinically useful in diseases in which neutrophilic inflammation plays a significant role (Mumaw et al., 2015).

A concise overview of these studies of dog and cat MSC secreted factors is presented in Table 5.

**TABLE 5:** Small companion animal MSC secretome components, targets, effects, and potential therapeutic uses.

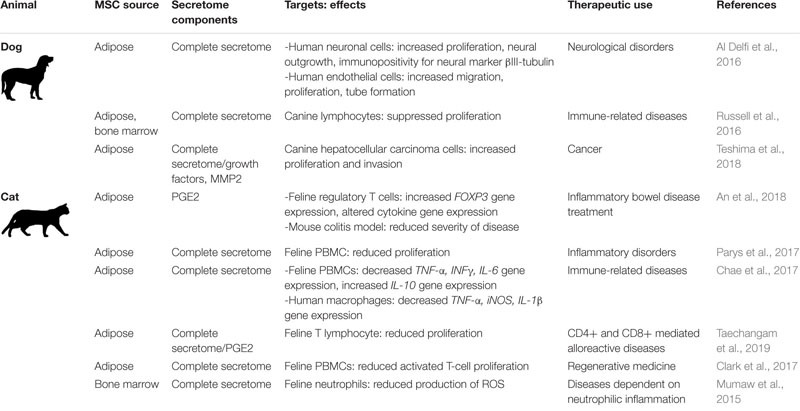

*IFN-γ, interferon-γ; IL-6: IL-10, interleukin-10; FOXP3, forkhead box P3; MMP2, matrix-metallopeptidase 2; PBMCs, peripheral blood mononuclear cells; PGE2, prostaglandin E2; ROS, reactive oxygen species; TNFα, tumor necrosis factor α.*

### Naïve Large Animal MSCs

Horse MSC isolation and therapeutic application for orthopedic injuries was first described in 2003 (Smith et al., 2003). The technique was declared “rational and feasible,” but conclusions could not be made based on a single case report without appropriate controls. More comprehensive studies, carried out in the past 15 years, have established that autologous MSC therapy is not detrimental to horses and the use of MSCs to treat orthopedic injuries has been accepted as a valuable therapeutic approach (Bogers, 2018; Al Naem et al., 2020). There are still many unanswered questions about the immunogenicity of allogenic horse MSC. Although allogenic MSC hold promise as treatments for numerous diseases of horses, such as endotoxemia, IBD, asthma and recurrent uveitis, more data are needed before allogenic cells can be used clinically (Berglund et al., 2017b; MacDonald and Barrett, 2020).

To the best of our knowledge, neither the cell-free complete secretome nor specific secretome components of horse MSCs have been delivered to horses *in vivo*. However, we and others have worked extensively *in vitro*, and to a lesser extent *in vivo* in rodent models, to characterize the horse MSC secretome and its effects on target cells (Table 6). Specifically, our research focuses on the horse peripheral blood (PB) derived-MSC secretome in the context of cutaneous skin wounds, which is not only of significant importance in equine medicine but can also greatly benefit human medicine by taking advantage of horse cutaneous wounds as translational models for the evaluation of human MSC-based therapies (Harman et al., 2019). For example, we found that *in vitro* (i) endothelin 1 (ET1), IL-8, platelet derived growth factor-AA (PDGF-AA), and insulin-like growth factor binding protein 2 (IGFBP2), present in the horse PB-MSC secretome promotes angiogenesis and (ii) plasminogen activator inhibitor 1 (PAI-1) and tenascin-C (TNC) secreted by PB-MSC increases fibroblast migration (Bussche and Van de Walle, 2014; Harman et al., 2018). Importantly, we confirmed the active roles of PAI-1 and TNC in fibroblast migration by repeating the experiments with secretome in which these factors were silenced using RNA interference and followed up by testing the contribution of these MSC-secreted proteins to wound healing *in vivo* in a mouse full-thickness skin injury model. Although this work confirmed the bioactive roles of these factors, our results showed that these two proteins did not account for the full wound healing effect of the complete secretome (Harman et al., 2018). Additional studies from our group identified anti-microbial peptides in the horse PB-MSC secretome that inhibit the growth of contaminating bacteria commonly found in skin wounds, as well as cysteine proteases that destabilize bacterial biofilms *in vitro* (Harman et al., 2017b; Marx et al., 2020). Moreover, we confirmed that the chemokine C-X-C motif ligand 6 (CXCL6) secreted by horse PB-MSC acts as a chemoattractant for neutrophils *in vitro* (Harman et al., 2020). Collectively, our research has identified specific factors secreted by PB-MSC that promote various aspects of skin wound healing, supporting the notion that the complete MSC secretome provides therapeutic benefits by targeting various aspects of specific disease processes.

**TABLE 6:** Horse MSC secretome components, targets, effects, and potential therapeutic uses.

**MSC Source**	**Secretome components**	**Targets: effects**	**Therapeutic use**	**References**
Peripheral blood	ET1, IL-8, PDGF-AA, IGFBP2	Endothelial cells: increased angiogenesis	Tissue regeneration	Bussche and Van de Walle, 2014
Bone marrow	Glycosaminoglycan	BM-MSC: decreased PDL	Maintaining stemness	Sasao et al., 2015
Peripheral blood	Complete secretome	Dermal fibroblasts: increased migration, altered gene expression	Cutaneous wound healing	Bussche et al., 2015
Amnion	Complete secretome, EV	LPS stimulated and unstimulated alveolar macrophages: cytokine secretion	Inflammatory lung diseases	Zucca et al., 2016
Peripheral blood	Antimicrobial peptides	*E. coli, S. aureus*: inhibition of growth, biofilm formation	Cutaneous wound healing	Harman et al., 2017b
Peripheral blood	Complete secretome	Dermal fibroblasts, healthy and dysregulated: alterations in morphology, proliferation, gene expression, contractile capacity and susceptibility to senescence	Fibroproliferative disorders	Harman et al., 2017a
Bone marrow	Complete secretome	Corneal stromal cells: increased migration	Corneal wound healing	Sherman et al., 2017
Bone marrow	Galectin-1/3	BMMSC: increased motility	Osteoarthritis	Reesink et al., 2017
Peripheral blood	PAI-1, tenascin-C	Dermal fibroblasts, mouse skin wounds: increased migration, wound closure	Cutaneous wound healing	Harman et al., 2018
Amnion	MicroRNAs	Secretome not tested with targets in a model system	Regenerative medicine	Lange-Consiglio et al., 2018
Adipose	EV derived small RNAs	Secretome not tested with targets in a model system	Regenerative medicine	Capomaccio et al., 2019
Bone marrow	Inflammatory, angiogenic proteins	Secretome not tested with targets in a model system	Osteoarthritis	Bundgaard et al., 2020
Peripheral blood	Complete secretome, cysteine proteases	*P. aeruginosa, S. aureus, S. epidermidis*: inhibition and destabilization of biofilms	Bacterial skin infections	Marx et al., 2020
Peripheral blood	CXCL6	Neutrophils: chemotaxis	Tissue repair	Harman et al., 2020

*BM-MSC, bone marrow-derived MSC; CXCL6, chemokine (C-X-C motif) ligand 6; ET1, endothelin 1; EV, extracellular vesicles; IGFBP2, insulin growth factor binding protein 2; IL-8, interleukin-8; LPS, lipopolysaccharide; NA, not applicable; PAI-1, plasminogen activator inhibitor 1; PDGF-AA, platelet derived growth factor-AA; PDL, population doubling level.*

Early studies of MSCs from cows, pigs, sheep, and goats, primarily focused on MSC characterization based on phenotypic marker expression and the potential to differentiate into adipocytes, chondrocytes, and osteocytes *in vitro* (Bosch et al., 2006; Cardoso et al., 2012; Heidari et al., 2013; Mohamad-Fauzi et al., 2015). More recently, global proteomic analysis of the secretome of cow endometrial MSCs identified 302 unique proteins, including those with anti-inflammatory or antibacterial properties and proteins related to tissue remodeling. After stimulating these MSCs with lipopolysaccharide (LPS), an increased 397 proteins were detected in the secretome, particularly those proteins involved in immunomodulation and tissue repair, leading the authors to conclude that these cow MSCs could be useful to treat reproductive diseases of cattle (de Moraes et al., 2017). Additional *in vitro* studies showed that the secretome from fetal cow MSC reduced the growth of *S. aureus* (Cahuascanco et al., 2019) and promoted endothelial cells to form tubules, an *in vitro* proxy for angiogenic potential (Jervis et al., 2019). Based on currently available data, the cow MSC secretome has been proposed as a treatment for mastitis, wound healing, nerve injuries, degenerative joint diseases and other diseases of the skeletal system, as well as diabetes mellitus (Gugjoo et al., 2019; Hill et al., 2019).

The immunomodulatory functions of soluble factors secreted by pig MSC have been studied *in vitro*, demonstrating that PGE_2_ suppresses the functionality of dendritic cells and T-cells (Khatri et al., 2015). Applying the secretome of pig corneal MSCs to injured corneal endothelial cells *ex vivo*, significantly reduced endothelial cell loss when compared to control conditions (Rouhbakhshzaeri et al., 2019). Moreover, it was found that LPS-damaged pig enteric ganglia were protected upon treatment with the secretome of pig MSC using an *in vitro* model of IBD (Dothel et al., 2019). The activity of pig MSC-derived EVs has also been studied in depth. For example, a study comparing the miRNA, RNA, and protein, expression profiles in the complete secretome of pig AT-derived-MSC to those profiles found in the EV fraction from these cells, showed that 4 miRNAs and 255 mRNAs were specifically enriched in EVs (Eirin et al., 2017). Another study evaluated the anti-influenza activity of EVs isolated from pig BM-MSC in lung epithelial cells *in vitro* and in a pig model of influenza infection *in vivo* (Khatri et al., 2018). The *in vitro* experiments showed that EVs were incorporated into epithelial cells, inhibited the hemagglutination activity and replication of influenza virus, and reduced virus-induced apoptosis of lung epithelial cells. *In vivo*, treatment with EVs significantly reduced influenza virus shedding in the nasal epithelium, viral replication in the lungs, and virus-induced proinflammatory cytokines in the lungs of infected pigs (Khatri et al., 2018).

Sheep MSCs have primarily been studied in terms of their potential to contribute to joint and cartilage repair (Music et al., 2018). For example, the administration of chondrogenically predifferentiated MSCs, embedded in hydrogels at the site of induced osteochondral injury in the medial femoral condyle of sheep, resulted in significantly improved histological scores at 6 months and 1-year post-administration when compared to controls (Zscharnack et al., 2010; Marquass et al., 2011). Since it is known that implanted MSC do not persist long term, these encouraging findings could be a result of factors secreted by the MSCs. To the best of our knowledge, however, specific effects of the sheep MSC secretome on osteochondral defects has not been examined *in vitro* nor *in vivo.*

### Manipulated Small Companion Animal MSCs (Dog)

Many strategies to optimize therapies with dog MSCs focus on enhancing the differentiation potential of these cells, primarily into chondrocytes and osteocytes. A main goal of establishing a stable chondrocyte phenotype from dog MSCs is to increase their deposition of articular cartilage proteins, so that these cells can become an effective treatment option for chronic OA. For example, it was found that exposing canine AT-MSCs to hypoxic conditions resulted in increased proliferation, a downregulation of genes associated with senescence like histone acetylase 1 (HDAC 1) and DNA-cytosine-5-methyltransferase (DNMT1), and an upregulation of genes that are associated with the potential to differentiate into chondrocytes like collagen type II alpha 1 (COL2A1) (Lee et al., 2016). Another study showed that culturing dog AT-MSCs with dimethyloxalylglycine (DMOG), which mimics hypoxic conditions by stabilizing hypoxia-inducible factor-1alpha (HIF1a), led to an increased expression of the signal protein vascular endothelial growth factor (VEGF), important in angiogenesis and thus, beneficial in diseases with ischemic conditions. However, high concentrations of DMOG did inhibit MSC proliferation (Kim S.M. et al., 2019). Importantly, it was found that the serum used in MSC cultures can alter the immunomodulatory properties of dog AT-MSC, since MSCs cultured in serum-free medium secreted lower levels of PGE_2_ and were less efficient in inhibiting interferon (INF)-γ secretion by activated T-cells (Clark et al., 2016). Culturing dog BM-MSC with pentonsan polysulfate (PPS) in a micromass culture system, successfully enhanced chondrogenesis and proteoglycan deposition. However, repeating these experiments in an alginate culture system did not result in a chondrocyte phenotype, pointing out the importance of the culture conditions for obtaining the desired MSC phenotype (Bwalya et al., 2017). Increased chondrogenesis and glycosaminoglycan (GAG) deposition was also observed when dog BM-derived peri-adipocytes (BMPAGs) were stimulated with fibroblast growth factor 2 (FGF2) in serum-free medium. BMPAGs are MSCs derived from cells adhering to adipocytes in the BM, and the authors proposed that this special site of isolation explained a lower donor variability in their results when compared to earlier studies that used BM-MSC (Endo et al., 2019). To increase clinical effectiveness of dog BM-MSC, Steffen et al. (2019) attached BM-MSC on a collagen microcarrier scaffold, in the presence or absence of immobilized TGF-ß1 and found an increased chondrogenic phenotype *in vitro*. Following up on this finding in a clinically study with canine patients suffering from intervertebral disc (IVD) degeneration, however, did not find any improvement that could be associated with the MSC treatment (Steffen et al., 2019).

While the majority of dog MSC studies focus on increasing differentiation potential, as outlined above, some studies did investigate the effects on paracrine signaling by priming MSCs. Dog umbilical cord blood (UC)-derived MSCs were primed with β-tricalcium phosphate, a combination previously found to produce promising osteogenic material (Jang et al., 2008), and then evaluated in an ectopic implantation model. On day 1 after implantation, tissue collected from UC-MSC-β-calcium implants showed an increase of *IL-1*, *IL-6*, and *VEGF* RNA expression as well as increased protein levels of IL-6 and VEGF when compared to controls, and this cytokine release was proposed to mediate the enhanced bone formation observed (Byeon et al., 2010). In addition to the potential of dog MSCs to modulate chondro- and/or osteogenesis, their benefits in immune-mediated diseases, such as inflammatory bowel syndrome that affects both dogs and humans, have also been explored (Hoffman and Dow, 2016). In this context, it was investigated how gastrointestinal microbes interact with dog AT-MSCs in order to understand if an altered gastrointestinal microbiome affects MSC therapy outcome in IBD (Kol et al., 2014). Based on the knowledge that (i) MSCs are known to express pattern recognition receptors (PRRs) and (ii) activation of MSCs through PRR ligands alters the MSC secretome (DelaRosa and Lombardo, 2010), it was explored whether co-culture of dog AT-MSCs with gastrointestinal commensal (*Lactobacillus acidophilus*) and pathogenic (*Salmonella typhimurium*) bacteria affected their phenotype. Although no increased cell death or upregulation of surface proteins major histocompatibility complex (MHC)-II, cluster of differentiation (CD)80/CD86, or CD1 was detected, the canine MSCs (i) did express higher RNA levels of *COX2, IL6* and *IL8*, (ii) secreted more PGE_2_, IL-6 and IL-8, and (iii) showed a higher ability to inhibit mitogen induced T-cell proliferation (Kol et al., 2014). The authors concluded that microbe-MSC interaction alters MSC functionality, and thus, that this needs to be taken into consideration when MSCs are explored as therapy in diseases associated with bacterial colonization (Kol et al., 2014). Another study evaluated the value of PRR expression for priming of AT-MSC in both mouse and dog models (Johnson et al., 2017). First, they showed that activation of mouse MSCs with poly I:C through the PRR Toll-like receptor 3 (TLR3) resulted in an altered secretome profile including increased secretion of the monocyte chemoattractant CC-chemokine ligand 2 (CCL2). CM from these primed MSCs (i) led to increased murine monocyte recruitment in an *in vitro* migration assay and (ii) stimulated neutrophils to increase their phagocytosis of bacteria *in vitro*. A follow up *in vivo* experiment in mice showed increased homing of activated MSCs to infected wounds (Johnson et al., 2017). When the effects of intravenously injected allogenic poly I:C-activated dog AT-MSCs were evaluated in canine patients suffering from chronic multi-drug resistant bacterial infections, the authors found that the MSC infusions were well tolerated, with no notable side effects, and that the conditions in all enrolled dogs either improved of resolved by the end of observation period. This prompted the authors to conclude that their pre-clinical study provides strong rationale to establish primed MSCs as a therapy for chronic bacterial infections (Johnson et al., 2017).

Due to their low immunogenicity and their homing ability, dog MSCs have also been explored as “trojan horses.” For example, the canine adenovirus ICOCAV17 has anti-tumor effects, but this virus is readily neutralized by the host immune system. To allow ICOCAV17 to reach the tumor site, dog AT-MSCs were infected with this virus and used to treat 27 dogs suffering from various cancerous diseases (Cejalvo et al., 2018). Of those, 74% benefited from the therapy and 14% even showed total remission. Interestingly, the study found increased immune cell infiltrations into the tumors after treatment, and this immune-related response to the infected MSCs was deemed to play an important role in the observed clinical benefits (Cejalvo et al., 2018). Similar studies in humans, using the human oncolytic adenovirus ICOVIR-5 to infect human MSCs, however, were less promising (Garcia-Castro et al., 2010; Melen et al., 2016; Ruano et al., 2020). When the cellular responses of human and dog MSCs to ICOVIR-5 and ICOCAV17, respectively, were compared, it was found that ICOVIR-5, but not ICOCAV17, intrinsically induces a strong phosphorylation of AKT and c-JUN (Rodríguez-Milla et al., 2020). Activation of the AKT pathway is associated with (i) virus latency by suppressing apoptosis of the host cell, (ii) host cell survival in chronic viral infections, and (iii) short-term cellular survival in acute viral infections depending on the virus and type of infection (Cooray, 2004). The authors concluded that an impaired cellular signaling in dog MSCs after ICOCAV17 infection, due to the lack of AKT activation, might lead to a more restricted host immune response after injection of dogs with these virus-infected MSCs, which could explain the better clinical outcome (Rodríguez-Milla et al., 2020). This study is a nice example of how comparative studies between species can lead to a better understanding of the underlying mechanisms responsible for certain clinical outcomes.

In addition to infecting dog MSCs with oncolytic viruses, several studies have been conducted to evaluate the use of genetically modified MSCs to treat cancer in dogs. For example, the effects of INF-β overexpression in dog AT-MSCs against canine melanoma has been studied intensively (Seo et al., 2011; Ahn et al., 2013; Han et al., 2015b). Transfected MSCs performed slightly better than naïve cells *in vitro*, with their secretome showing improved pro-apoptotic and cell growth inhibitory effects on canine melanoma cells (Han et al., 2015b). *In vivo*, INF-β overexpressing dog AT-MSCs did migrate to the tumor site after subcutaneous injection in a mouse model, and these mice showed increased survival time when the MSC treatment was combined with the chemotherapeutic cisplatin (Seo et al., 2011). These findings were corroborated a few years later when INF-β overexpressing dog AT-MSCs were used *in vitro* and in a xenograft *in vivo* mouse model of canine melanoma. Importantly, this study found that melanoma cell proliferation was inhibited using an indirect co-culture system, indicating that the anti-tumor effects rely on factors in the MSC secretome (Ahn et al., 2013). Genetically modified dog AT-MSCs overexpressing cytotoxic T-lymphocyte antigen 4 (CTLA4), with the goal to increase immune suppressive properties, have also been explored. Specifically, T-lymphocyte infiltration into thyroid glands was found to be decreased, as well as thyroglobulin antibodies in the serum, upon treatment with CTLA4-AT-MSCs in an induced thyroiditis model in beagles (Choi et al., 2015). These cells have also been used as treatment in a case study of a dog with therapy-resistant pemphigus foliaceus, an immune-mediated disease that leads to severe skin lesion and reduces quality of life immensely (Olivry, 2006). In this case study, the dog received 21 injections with CTLA4-AT-MSCs over a period of 20 months. The lesions improved and the patient reached a stable state of disease that could be controlled with low-dose prednisolone for a year (Han et al., 2015a). Lastly, dog AT-MSCs transduced with a tyrosine mutant adeno-associated virus 2 vector to overexpress stromal-derived factor-1 (SDF-1), with the goal to promote MSC homing and survival, have been evaluated in dogs with dilated cardiomyopathy (Pogue et al., 2013). Although the SDF-1-AT-MSCs were successfully administered via retrograde coronary venous delivery and no adverse effects were observed, the treatment failed to improve clinical outcome in the enrolled dogs (Pogue et al., 2013).

### Manipulated Large Animal MSCs (Horse)

As previously mentioned, the horse is a widely used and well-accepted model for OA. Early on, the hypothesis was that MSCs would engraft in pathological joints and differentiate into chondrocytes, and as such, mitigate joint trauma. In order to increase clinical outcome, horse BM-MSCs were primed *in vitro* with TGF-β1 and insulin-like growth factor-I (IGF-I), which increased the chondrogenic potential of these horse MSCs (Worster et al., 2001). However, it is now generally accepted that the observed therapeutically effects of MSCs are not due to their engraftment, but due to immunomodulation via paracrine signaling. Although it is known that the expression of immunogenic and immunomodulation-related genes and molecules in MSCs change in a proinflammatory environment, potentially activating the immunomodulatory properties of these cells, it was found that the equine synovial fluid of inflamed joints alone was not sufficient to enhance the immunoregulatory profile of horse BM-MSCs (Barrachina et al., 2016). Consequently, several studies focused on increasing the immunomodulatory properties by priming horse MSCs *in vitro* in culture before administration *in vivo*. For example, a dose-dependent stimulation of horse BM-MSCs with the cytokines tumor necrosis factor α (TNF-α) and INF-γ led to an upregulation of immunoregulatory genes without affecting viability and differentiation potential (Barrachina et al., 2017a, 2018). Although INF-γ priming increased the chondroprotective effect of horse BM-MSCs, the expression of MHC-I and MHC-II was also upregulated, implicating an increased immunogenicity (Barrachina et al., 2017b; Hill et al., 2017; Cassano et al., 2018). In line with these *in vitro* results, an *in vivo* study in an equine OA model showed only slightly improved clinical signs, as well as synovial inflammatory signs, when horses were treated with allogeneic naïve or TNF-α/INF-γ primed horse BM-MSCs. Moreover, injection of these primed MSCs led to a transient local inflammation reaction after the second injection, most likely due to the production of allo-antibodies that recognized these primed MHC-mismatched MSCs with high expression levels of MHC-class I and II molecules (Berglund and Schnabel, 2017; Barrachina et al., 2018, 2020). More recently, priming horse BM-MSCs with TGF-β2 has been identified as a promising strategy to inhibit INFγ-induced MHC I and II surface expression *in vitro*, thus, potentially improving MSC survival and therapeutic efficacy (Berglund et al., 2017a).

Genetically modified horse MSCs have also been explored and one of the first *in vivo* studies used horse BM-MSCs that were successfully transduced with an adenoviral vector to overexpress IGF-I in a model of equine tendinitis (Schnabel et al., 2009). Tendon histological scores improved after treatments with both naïve MSCs and IGF-I-MSCs, leading to the conclusion that horse MSCs might be beneficial for the treatment of tendinitis, but without a superior effect from transfected MSC (Schnabel et al., 2009). In equine OA, the nuclear factor κB (NFκB) signaling pathway, which can be activated by the cytokines IL-ß1 and TNF-α that are naturally present in inflamed joints, has been determined to be a key signaling pathway contributing to disease pathology. IL1-β and TNF-α not only activate NFκB but become in turn also upregulated by this activated pathway, thus creating a positive autoregulatory loop that can amplify inflammation (Marcu et al., 2010). Equine MSCs have been engineered with the goal to interrupt this inflammatory response. For example, the usefulness of a tunable gene expression vector under the control of an NFκB-responsive enhancer/promoter that can be regulated by the pro-inflammatory cytokines IL-1β and TNF-α has been explored (Gabner et al., 2016). As proof of concept, the reporter gene luciferase was used to show that stimulation of transduced MSCs with IL-1β and TNF-α indeed led to the expression of the reporter gene in a dose dependent manner (Gabner et al., 2016). In a follow-up study, the authors then replaced the reporter gene with the gene encoding for interleukin-1 receptor antagonist (IL-1Ra) and found that its expression could be modulated by repeated cycles of induction with TNF-α. Importantly, they could demonstrate that IL-1Ra present in the secretome of these transduced MSCs effectively blocked OA onset in an *in vitro* model using horse chondrocytes (Gabner et al., 2018). Based on these findings, the authors suggested that transduced MSCs that are administered to inflamed joints and express tunable IL-1Ra in response to the pro-inflammatory cytokines present in these inflamed joints, are a promising strategy to promote joint homeostasis.

## Discussion

Understanding the biologically active factors that make up the human MSC secretome and manipulating these cells to consistently secrete factors of therapeutic importance, will improve MSC secretome-based therapies. Emerging single-cell technologies will undoubtedly help decipher the heterogeneity of MSCs and allow for the selection of MSC subsets that secrete therapeutically desirable factors. To date, single-cell transcriptomic analyses of human MSCs resulted in varied outcomes. For example, umbilical cord-derived MSCs were found to exhibit limited heterogeneity, whereas Wharton’s jelly derived MSCs were found to be functionally heterogeneous in terms of proliferative capacity and wound healing potential (Huang et al., 2019; Sun et al., 2020). Single-cell RNA sequencing of mouse BM-derived MSCs revealed multiple profiles as well, some associated with distinct differentiation potential (Freeman et al., 2015). Our group used single-cell transcriptomics to analyze donor-matched equine MSCs isolated from three different tissue sources and we found inter- and intra-source genetic heterogeneity that resulted in functional heterogeneity in immune function and cell motility (Harman et al., 2020). The emerging technology of high-resolution precision proteomics is currently only being used to evaluate cancer cellular heterogeneity (Waas and Kislinger, 2020), but will certainly be transferrable to MSCs, where this technique can provide additional insights into the heterogeneity of MSC populations to allow for the purification of MSC subpopulations with high secretory potential.

In addition to identifying the molecules produced by MSCs that have the functional characteristics to lead to desired clinical outcomes, there are further aspects to consider when moving MSC secretome therapy from bench to bedside. Here, we discuss two of those aspects by asking the following questions. First, is the use of a rich compilation of bioactive MSC secreted factors required for maximal therapeutic benefit, and second, what options are available for delivery of the MSC secretome to target tissues?

### Advantages and Disadvantages of Using a Compilation of Bioactive MSC Secreted Factors

The use of a compilation of bioactive factors secreted by MSCs that have either been primed to overproduce therapeutically valuable molecules or genetically engineered to produce and secrete these molecules, may be more effective than solely administering the individual factors of interest. As discussed earlier, studies from our group indicated that although specific proteins in the horse PB-MSC secretome contribute to cellular functions associated with wound healing, they did not account for the full effectiveness of the complete secretome as observed in both *in vitro* and *in vivo* wound healing assays (Harman et al., 2018). In general, most studies documenting the efficacy of the MSC secretome to promote tissue repair and/or modulate the immune system do not indicate precisely which factors are responsible for the beneficial effects. The most obvious reason for using a compilation of bioactive MSC secreted factors over discrete individual factors, is the fact that the secretome is comprised of a myriad of bioactive nucleic acids, proteins, and lipids, that all have the potential to interact with target cells and tissues on different levels (Harrell et al., 2019). Consequently, the use of a compilation of MSC secreted bioactive factors provides numerous molecules that may function together in networks in order to obtain the maximal effect. This is illustrated by previously discussed data from our own group. We have identified proteins with regenerative properties and proteins with antimicrobial properties in the secretome of horse PB-MSCs (Bussche and Van de Walle, 2014; Bussche et al., 2015; Harman et al., 2017a,b, 2018, 2020, Marx et al., 2020). Using these identified factors individually could be therapeutically useful to promote wound healing or to fight bacterial infections. However, using the secretome as a whole may capitalize on a treatment that reduces bacteria in infected wounds, while simultaneously restoring the tissue damage caused by acute injury and pathogens.

This benefit is also evident by the recognition that MSC-derived EVs, widely studied as a form of cell-free MSC therapy, are made up of a compilation of factors, including nucleic acids, particularly small regulatory RNAs, proteins and lipids. Secreted factors contained in EVs are more stable than secreted factors that are free in solution and they are more likely to be taken up by target cells via interactions of surface ligands/receptors, adhesion of membrane integrins, or endocytosis of the EVs (Sarko and McKinney, 2017; Eleuteri and Fierabracci, 2019). EVs from many cell types are known to be involved in cell-cell signaling, as well as tissue regeneration, and it has been demonstrated that EVs are comprised of RNAs, proteins, and lipids, that are distinct from those secreted freely from their cells of origin (Barreca et al., 2020). For example, comparative analyses of miRNAs detected in EXOs and the EXO-cells-of-origin have clearly demonstrated that miRNA composition of EXOs and the cells they were secreted from differ widely, suggesting active packaging of miRNAs into this class of EV (Zhang et al., 2015). Among miRNAs known to be upregulated in EVs, are those involved in the regulation of angiogenesis (Liang et al., 2016; Salinas-Vera et al., 2018). Moreover, EVs transport various cytosolic proteins involved in cell proliferation and migration, such as FGF2 that lacks the exocytosis signals needed to be secreted through the endoplasmic reticulum-Golgi pathway (Candela et al., 2010). In general, EVs are enriched in sphingosine-1-phosphate, a signaling lipid that in itself induces cell proliferation and migration (Xiang et al., 2018).

In addition to the important therapeutic benefit of administering a compilation of bioactive factors secreted by MSCs, there are certainly some disadvantages to this approach. Most notably, and as discussed throughout this review, is the inconsistency in the effectiveness of secretome therapy due to variability of the MSC secretome based on individual donor, tissue source of origin, culture method, and duration of MSCs in culture. As reviewed, these inconsistencies can be addressed by priming and/or genetically modifying MSCs to generate a more consistent secretome. Other strategies to produce a more consistent MSC secretome include (i) consideration of the age, sex and health status of MSC donors, (ii) purposefully using MSC from specific tissue sources, (iii) carefully documenting MSC culture conditions and (iv) limiting the length of time MSCs are maintained in culture (Sagaradze et al., 2018). Indeed, experts in the field of MSC secretome-based drug development state that generating a consistent MSC secreted product with testable potency is the first step needed to move this cell-free therapy from laboratory testing to clinical use (Sagaradze et al., 2018).

### Delivery of the MSC Secretome

As described in this review, the secretomes of MSCs isolated from various species and/or different tissue sources contain bioactive factors that have the potential to be used as cell-free therapies. In order to be effective, MSC secretome components must be delivered to target tissues and interact with target cells. On the one hand, administering the MSC secretome therapeutically avoids some of the hurdles associated with MSC cell-based therapies such as the risk of triggering the innate or adaptive immune responses and the possibility of donor cell engraftment and tumorigenicity (Caplan et al., 2019). On the other hand, MSC secretome-based therapies presents challenges such as retaining secreted factors at the appropriate sites and protecting them from degradation. Select strategies for the delivery of the stem cell secretome have been recently reviewed (Daneshmandi et al., 2020), so here, we will primarily discuss additional strategies that have been described for the delivery of the secretome from other cell types that may also be appropriate for administering MSC secreted factors. Modes of secretome administration can be roughly divided into two categories: direct and associated with a delivery vehicle.

Direct administration includes the injection or application of CM from cultured MSCs at the site of injury, e.g., cutaneous wounds, as well as the injection of exosomes (EXOs) systemically into the blood stream (Daneshmandi et al., 2020). The MSC secretome may also be delivered directly by inhalation for certain diseases (Khan et al., 2017; Grinblat et al., 2018; Dane et al., 2020). For example, treatments with CM from human induced pluripotent stem cells (iPSCs) were delivered by inhalation every 5 days following unilateral pneumonectomy (PNX) in a dog model of destructive lung disease. This study revealed that repetitive inhalation of the iPSC secretome increased alveolar angiogenesis and enhanced septal remodeling associated with improved gas exchange compensation in the lungs (Dane et al., 2020). Moreover, intranasal delivery of the MSC secretome may also have neuroprotective effects, as shown in a study evaluating the efficacy of the secretome of the human amnion-derived multipotent progenitor cells, named ST266 (Grinblat et al., 2018). Specifically, five daily intranasal treatments with ST266 of mice with surgically induced optic nerve crush injuries, resulted in increased retinal ganglion cell (RGC) survival and showed a trend toward reduced RGC axon and myelin damage (Grinblat et al., 2018). In another study, intranasally administered ST266 showed potent neuroprotective and anti-inflammatory effects on the optic nerve in a mouse experimental autoimmune model of multiple sclerosis (Khan et al., 2017).

Historically, MSCs themselves have been used as “delivery vehicles” for their secretome which evolved from the practice of injecting MSCs, locally or systemically, with the goal of having them differentiate and expand at the site of injury to replace damaged tissue (Galipeau and Sensébé, 2018; Krueger et al., 2018; Martin et al., 2019). As it became clear that MSCs do not survive long after administration and that the beneficial effects they exert are due to the factors they secrete that influence recipient target cells, MSCs have served as delivery vehicles in their own right (Spees et al., 2016; Krueger et al., 2018). An active field of research is the engineering of delivery vehicles that allow for the prolonged release of therapeutic molecules at sites of injured tissue. Cells that secrete bioactive factors or bioactive factors in solution can be carried in the vehicles. Delivery vehicles include (i) synthetic polymer-based scaffolds, which increase hydrophilicity and improve cell/secretome immobilization, (ii) hydrogels that retain cells or secreted factors and allow for controlled release, and (iii) fabricated secretome-loaded microparticles that can reside in damaged tissues releasing bioactive factors for days without being rejected by the recipient immune system (Ryu et al., 2019; Daneshmandi et al., 2020). Our group conducted an *in vitro* proof-of-concept study aimed to determine if equine PB-MSCs survived when encapsulated in core shell hydrogel microcapsules, and if MSC secreted factors could diffuse through the capsules and affect target cells (Bussche et al., 2015). We found that MSCs survived for over 3 weeks in the capsules and that CM collected from these encapsulated MSCs promoted dermal fibroblast migration and changes in fibroblast gene expression, suggesting that MSCs encapsulated in this way may be appropriate for therapy (Bussche et al., 2015). Commercially available, implantable cell devices may also serve as delivery vehicles for the MSC secretome. In a rat model of myocardial infarction, animals were subjected to permanent ligation of the left anterior descending coronary artery and then treated with a subcutaneous implantation of human cardiac stem cells enclosed in a TheraCyte device (Kompa et al., 2020). This device retained the cells and protected them from the recipient immune system, while allowing cellular secreted factors to exit. Treated rats showed preserved cardiac function, reduced fibrotic scar tissue, interstitial fibrosis and cardiomyocyte hypertrophy, as well as increased myocardial vascular density when compared to controls (Kompa et al., 2020).

Taken together, both the direct and indirect methods shown to be effective at delivering the secretomes of other cells types are highly likely translatable to the MSC secretome as well. Such methodologies can then be tested and refined in translation animal models, as discussed above, to further improve and optimize MSC secretome therapy in humans.

## Conclusion

To optimize the human MSC secretome as a therapy, MSC-secreted factors must be better characterized and MSC cultures should be consistent in terms of reliably producing adequate quantities of biologically active molecules of interest. Methods to minimize variation between MSC cultures and to promote enhanced or selective secretion of specific bioactive molecules include priming and genetic engineering of MSCs. Once secretome components are optimized for maximum therapeutic benefits, targeted delivery methods are needed to direct them to injured tissues. Studies in veterinary species have already provided a wealth of information on which molecules are present in the MSC secretome and are therapeutically valuable, and on how to manipulate MSCs to preferentially secrete molecules of interest. Importantly, many of these studies have been carried out *in vivo* in these translational animal models with similar diseases as seen in humans, and they will continue to serve as valuable models to evaluate effective MSC-secreted factor delivery methods in a preclinical setting, that will not only benefit the model species, but human medicine as well.

## Author Contributions

RH and CM performed the bibliographic research, drafted the manuscript, and created the figures and tables. GV supervised the process, revised the manuscript, and finalized the manuscript. All the authors contributed to the article and approved the submitted version.

## Conflict of Interest

The authors declare that the research was conducted in the absence of any commercial or financial relationships that could be construed as a potential conflict of interest.
